# Exploring the spatiotemporal patterns of county-scale PM2.5 drivers in Shandong Province from 2000 to 2020

**DOI:** 10.1371/journal.pone.0310190

**Published:** 2024-10-03

**Authors:** Dongchao Wang, Xichun Li, Xinrong Duan, Huimin Yang, Baolei Zhang

**Affiliations:** 1 College of Geography and Environment, Shandong Normal University, Jinan, Shandong, China; 2 Inspur Software Technology Co., Ltd., Jinan, Shandong, China; 3 Shandong Provincial Institute of Land Surveying and Mapping, Jinan, Shandong, China; 4 Jinan Geotechnical Investigation and Surveying Research Institute, Jinan, Shandong, China; Jinan University, CHINA

## Abstract

In the rapid development of air pollution over the past two decades in Shandong Province, it has played a detrimental role, causing severe damage to regional ecological security and public health. There has been little research at the county scale to explore the spatiotemporal causes and heterogeneity of PM2.5 pollution. This study utilizes a Geographically and Temporally Weighted Regression Model (GTWR) to environmentally model meteorological elements and socioeconomic conditions in Shandong Province from 2000 to 2020, aiming to identify the key driving factors of PM2.5 concentration changes across 136 counties. The results show that PM2.5 pollution in Shandong Province peaked in 2013, followed by a rapid decline in pollution levels. Geographically, counties in the western plains of Shandong generally exhibit higher pollution levels, while most counties in the central hills of Shandong and the Jiaodong Peninsula are in low pollution areas. Strong winds positively influence air quality in the southeast of Shandong; high temperatures can ameliorate air pollution in areas outside the southeast, whereas air pressure exhibits the opposite effect. Precipitation shows a significant negative correlation in the Laizhou Bay and central Shandong regions, while relative humidity primarily exerts a negative effect in coastal areas. The impact of fractional vegetation cover is relatively mild, with positive effects observed in southern Shandong and negative effects in other regions. Population density shows a significant positive correlation in the western plains of Shandong. Economic factors exhibit predominantly positive relationships, particularly in the northwest and the Jiaodong Peninsula. Electricity consumption in southern Shandong correlates positively, while industrial factors show positive effects province-wide. PM2.5 pollution in Shandong Province demonstrates significant spatiotemporal heterogeneity, aligning with governmental expectations for the effectiveness of air pollution control measures. The conclusions of this study can be utilized to assess the efficiency of air pollution abatement at the county level and provide quantitative data support for the revision of regional emission reduction policies.

## 1. Introduction

The application of geospatial and temporal analysis techniques in investigating PM2.5 (particulate matter less than or equal to 2.5 micrometers) has significant importance in research [1]. PM2.5 particles are a major component of air pollution, capable of persisting in the atmosphere due to their small size and suspended nature, thus exerting widespread and profound impacts on human health and the environment. Environmental remote sensing provides a means of large-scale, real-time, continuous monitoring of PM2.5 concentrations [2]. These data can be used for formulating environmental policies [3], and exploring the transport and distribution of PM2.5 in the atmosphere [4]. Ground-based PM2.5 monitoring networks are limited by spatial coverage and maintenance costs, making them unsuitable for large-scale, long-term studies, often requiring supplementation with other data sources such as satellite remote sensing [5]. The global PM2.5 dataset of the Atmospheric Composition Analysis Group (ACAG) can be applied in various fields including environmental science [6], public health [7], and ecological restoration [8]. This dataset provides data support for researchers, policymakers, and the general public, aiding in better understanding and addressing atmospheric pollution issues.

Research into the driving factors of PM2.5 pollution is gaining popularity in academic circles. Meteorological elements, socioeconomic conditions, and Fractional Vegetation Cover (FVC) are key indicator parameters for PM2.5 driving models [9]. Meteorological elements (such as temperature, precipitation, wind speed, etc.) have significant impacts on PM2.5 concentration [10]. Atmospheric pressure features indirectly influence the spatial distribution of PM2.5 by altering atmospheric patterns. Studies have found a strong correlation between socioeconomic factors and air pollution, with an N-shaped Environmental Kuznets Curve (EKC) connection existing in developed regions [11]. Additionally, different socioeconomic factors contribute differently to air pollution, with industrial electricity consumption having the greatest impact on PM2.5 concentration [12]. Geospatial statistical tools have discovered a U-shaped EKC hypothesis, demonstrating the potential impact of socioeconomic factors on PM2.5 concentration [13]. As an important ecological indicator, FVC is used to assess surface vegetation conditions and is correlated with PM2.5 concentration [14].

Various methods have been employed to identify parameters influencing PM2.5 pollution, including Generalized Additive Models (GAM) [15], the expanded STIRPAT Models [16], and Geographic Detector Models [17]. Panel data, due to its advantages over traditional time series data or cross-sectional datasets, has been widely used by scholars to address PM2.5 concentration issues [18]. Similar methods can be used to discuss the impacts of anthropogenic and meteorological variables on PM2.5 pollution. The Logarithmic Mean Divisia Index (LMDI) method can detect the main factors causing changes in air pollution emissions, which is suitable for large-scale studies with long time series [19]. Additionally, subsequent dynamic spatial panel models provide a unique perspective for quantitatively studying the driving factors of PM2.5 concentration [20]. Three-dimensional chemical transport models have opened up avenues for revealing the mechanisms by which meteorological factors influence air quality [21]. Geographic Detectors and Spatial Autoregressive Models can explore the spatial variation patterns of PM2.5 driving factors [22], helping scholars understand the spatial morphology of the relationship between influencing factors and air pollution.

Recent studies have made significant contributions to effectively exploring the patterns of atmospheric pollution. However, most studies insist on using global regression techniques [23], ignoring the spatiotemporal variability of PM2.5 concentrations and their influencing factors. Traditional regression models generally assume parameter homogeneity globally, primarily serving to explore the overall relationship between dependent and independent variables. In short, the influencing factors of PM2.5 exhibit discontinuous, unstable heterogeneous fluctuations in both time and space, characteristics that classical regression methods inherently cannot express [24]. To mitigate the ecological crisis and public safety issues caused by air particulate pollution, environmental management departments need to comprehensively consider the different regional conditions and development statuses of counties and districts, devising diverse and targeted prevention and control measures to prevent pollution control policies from encountering "local resistance" [25]. Furthermore, research has found that pollutants emitted by social production activities are significantly affecting PM2.5 concentration distribution [26]. Our understanding of the causes of issues such as how pollutants diffuse in the atmosphere and the impacts of other factors on the diffusion process remains unclear [27]. Therefore, to clearly understand the spatiotemporal variation patterns of PM2.5 driving factors, it is necessary to introduce more effective spatiotemporal statistical tools, rigorously analyze the correlation between natural factors, socioeconomic factors, etc., and PM2.5 levels.

This paper focuses on the county scale of Shandong Province, utilizing advanced geospatial statistical tools to explore the driving factors of PM2.5 from the perspectives of sources, emission, and diffusion. We employ the Geographically and Temporally Weighted Regression Model (GTWR) [28] to quantify the spatiotemporal evolution patterns of key driving factors of PM2.5 in both time and space. The organization of this research is as follows: The data materials and research methods associated with this study are elaborated in detail in Section 2. The results and preliminary analysis of this study are presented in Section 3. Suggestions based on this research and further discussion are provided in Section 4. Finally, we summarize the findings in Section 5.

## 2. Materials and methods

### 2.1 Study area

**Fig 1:**
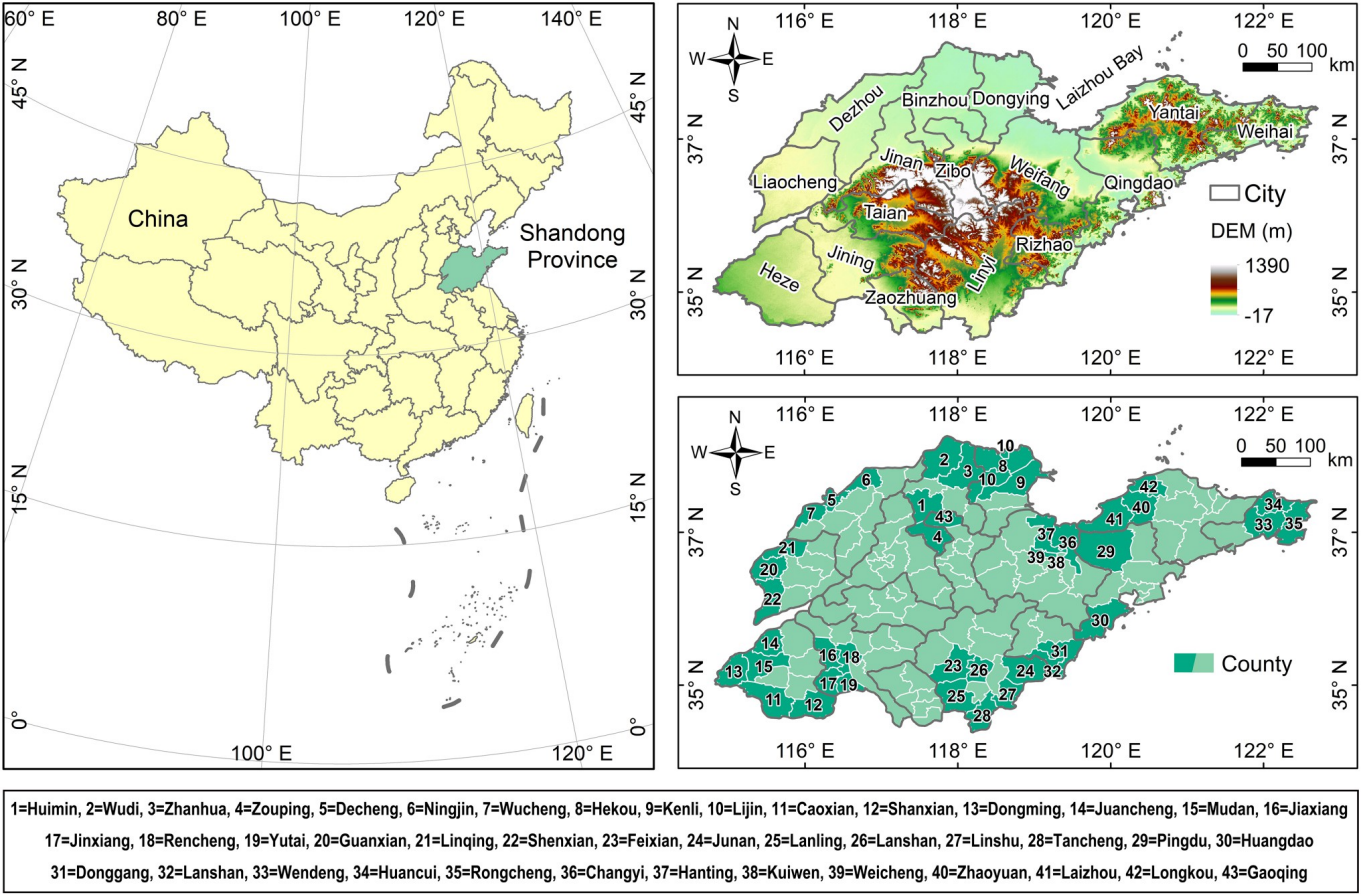
Topographical features and administrative divisions of Shandong Province, China.

Shandong Province is situated in the eastern coastal region of China, occupying a significant portion of the North China Plain, comprising 16 cities and 136 counties (Fig 1). The terrain slopes from east to west, with mountainous regions in the east and plains in the west, while the central part consists of hilly areas. It falls under a warm temperate monsoon climate, characterized by hot and rainy summers and cold and dry winters. Socially, rural areas constitute the majority, while urbanization is progressing rapidly, leading to significant urban-rural disparities [29]. Shandong Province consistently ranks as the second most populous province in China and holds a prominent position in terms of Gross Domestic Product (GDP), making it a crucial contributor to China’s economy and culture. Its economy is dominated by manufacturing and service industries, with well-developed agriculture and animal husbandry sectors. With an extensive coastline, Shandong Province shows promising development prospects in marine economy, fisheries, and marine technology. In summary, Shandong Province exhibits rich geographical, cultural, social, and economic characteristics, exerting significant influence on China’s economic and cultural development [30].

The meteorological, natural and socioeconomic explanatory variables in the Shandong Province show significant spatial non-stationary characteristics. Among the meteorological factors, precipitation decreases from southeast to northwest; air pressure varies with altitude and seasonal changes; relative humidity is high in coastal areas; temperature decreases from south to north and is lowest in coastal areas; and wind speed is higher along the coast than inland. In the natural landscape, vegetation cover is mainly concentrated in the central mountainous and coastal areas. Population and GDP are closely related, mainly in provincial capital and coastal metropolitan areas; electricity consumption coincides with economically developed areas; SO2 emissions are concentrated in inland areas with developed industries.

Air quality in Shandong Province exhibits spatial variability and interannual variations influenced by factors such as meteorological conditions, socioeconomic conditions, and distribution of pollutant sources. Its northern proximity to the Beijing-Tianjin-Hebei Economic Zone and western adjacency to Shanxi, a major coal-producing province, facilitate the inflow of exogenous PM2.5. Furthermore, frequent large-scale dust storms in northwest China can easily invade the North China Plain region [31], significantly affecting PM2.5 concentrations in Shandong Province.

### 2.2 Data sources

In the field of air pollution research, obtaining large-scale and long-term series of air pollution remote sensing data is crucial for exploring the spatiotemporal evolution patterns of air pollution. Commonly used air quality monitoring data from satellites such as MODIS, Landsat, and Sentinel provide extensive remote sensing datasets, greatly facilitating communication and collaboration in air pollution research. However, existing remote sensing data sources vary significantly in spatial resolution, with low-resolution data diminishing the applicability and quality of high-resolution data [32].

#### 2.2.1 Satellite-derived PM2.5

High-precision PM2.5 concentration data for Shandong Province were recalculated from the global PM2.5 dataset developed by the ACAG at the Washington University (https://sites.wustl.edu/acag/datasets/surface-pm2-5/) [33]. This dataset is available at both annual and monthly temporal resolutions for the period 1998–2022, with a spatial resolution of 0.01°×0.01° grid. It integrates Aerosol Optical Depth (AOD) data from satellite sensors with the GEOS-Chem chemical transport model and then calibrates the raw data with global surface observation data through Geographically Weighted Regression (GWR), resulting in a high-precision global PM2.5 dataset. Spatial analysis tools for zonal statistics were employed to analyze the average PM2.5 concentrations in each county of Shandong Province during 2000–2020.

#### 2.2.2 Meteorological factors

Meteorological factors, including precipitation, atmospheric pressure, relative humidity, temperature, and wind speed, were derived from reanalyzed data from Chinese surface meteorological stations (http://data.cma.cn/site). To interpolate discrete station data across the entire study area, an inverse distance weighted spatial interpolation method was utilized to produce a meteorological raster dataset with a spatial resolution of 0.01°×0.01°. Parameter indices include annual average precipitation (PREC), annual average atmospheric pressure (PRES), annual average relative humidity (RH), annual average temperature (TEMP), and annual average wind speed (WS). Subsequently, with the aid of spatial zonal statistics tools, the average meteorological factor data for each county in Shandong Province were analyzed (refer to Table 1). The Variance Inflation Factor (VIF) between the meteorological factors and the dependent variable was measured, and all the values were less than 7.5. It indicates that there is no significant multicollinearity between the meteorological elements, which did not interfere with the stability and interpretability of the regression coefficients in the regression model.

**Table 1:** Statistical results of meteorological factors from 2000 to 2020.

Variable	Min	Mean	Max	Standard deviation	VIF
PM2.5 (μg/m^3^)	19.85	66.21	110.25	13.27	
PREC (mm)	341.70	672.79	1386.90	151.39	1.21
PRES (kPa)	91.21	100.38	102.32	1.34	1.49
RH (%)	61.51	83.09	97.83	0.56	1.64
TEMP (°C)	8.33	13.74	16.16	1.00	2.42
WS (m/s)	1.09	2.68	7.11	0.86	1.44

#### 2.2.3 Natural and socioeconomic conditions

In addition to meteorological variables, this study selected FVC [34] from natural conditions and population density (POP) [35], GDP density (GDP) [36], electricity consumption density (EC) [37], and SO2 emissions (SO2) [38] from socioeconomic conditions as non-meteorological variables. Statistical results of natural and socioeconomic variables from 2000 to 2020 are presented in Table 2. All the values of VIF between natural-socioeconomic conditions and PM2.5 are less than 7.5, which indicates that there is no significant multicollinearity between the independent variables. The independent effect of each explanatory variable on the dependent variable can be easily isolated when the correlation of the variables is low.

**Table 2:** Statistical results of natural and socioeconomic variables from 2000 to 2020.

Variable	Min	Mean	Max	Standard deviation	VIF
PM2.5 (μg/m^3^)	19.85	66.21	110.25	13.27	
FVC (%)	2.00	66.82	93.00	11.78	1.96
POP (Person/km^2^)	0.81	613.71	2397.70	826.44	2.50
GDP (10^6^*Yuan/km^2^)	0.00	21.72	89.76	6.15	2.11
EC (10^6^*Wh/km^2^)	0.00	1580.72	28514.73	3635.77	2.97
SO2 (μg/m^3^)	0.41	43.97	105.57	21.18	2.40

FVC significantly influences PM2.5 concentrations, due to the high FVC can reduce PM2.5 concentrations through various mechanisms. Firstly, vegetation effectively intercepts and adsorbs particulate matter in the atmosphere, thereby reducing PM2.5 concentrations. Secondly, vegetation transpiration increases atmospheric humidity, promoting particle deposition and wet deposition, thus lowering PM2.5 concentrations. Additionally, vegetation absorbs atmospheric carbon dioxide through photosynthesis, promoting vegetation growth and ecosystem stability, indirectly reducing the number and intensity of PM2.5 emission sources. The increase in FVC plays a crucial role in improving air quality and reducing PM2.5 concentrations [39].

Population density, GDP density, and electricity consumption density in socioeconomic factors significantly impact PM2.5 concentrations. High population density is often closely associated with industrialization and urbanization, resulting in more emissions sources and anthropogenic activities, thereby increasing PM2.5 emissions and concentrations. High GDP density typically reflects the intensity of economic activities and energy consumption levels, with associated emissions (such as industrial emissions, vehicular emissions, etc.) increasing particulate matter concentrations in the atmosphere. Moreover, high electricity consumption density usually implies more energy utilization and production activities, including combustion of fossil fuels, releasing a large amount of air pollutants directly affecting PM2.5 concentrations [40]. Therefore, socioeconomic factors such as population density, GDP density, and electricity consumption density have significant impacts on increasing PM2.5 concentrations, necessitating corresponding measures in urban planning, industrial production, and energy utilization to reduce atmospheric pollution levels [41].

Emissions of sulfur dioxide (SO2) from social and industrial activities significantly affect PM2.5 concentrations. SO2 emissions lead to an increase in sulfur oxides in the atmosphere, thereby promoting the formation and increase of PM2.5. SO2 reacts with oxygen and water vapor in the atmosphere to form sulfuric acid droplets, one of the main components of PM2.5. These droplets serve as condensation nuclei, promoting the growth and coalescence of other particles, thereby increasing PM2.5 concentrations. Additionally, SO2 emissions participate in photochemical reactions to produce sulfuric acid droplets and sulfate particles, further increasing the quantity and concentration of PM2.5. Therefore, SO2 emissions play a significant role in increasing PM2.5 concentrations, necessitating effective measures to reduce SO2 emissions to improve atmospheric environmental quality [42].

### 2.3 Methodology

This study begins at the county level, utilizing satellite remote sensing datasets to estimate PM2.5 concentrations and employing spatial autocorrelation and GTWR methods to explore the spatiotemporal patterns of PM2.5 and its driving factors. Initially, utilizing zonal statistical tools, PM2.5 concentration distributions in Shandong Province were computed based on global PM2.5 products. Subsequently, several explanatory variables related to PM2.5 were selected as independent variables for spatial regression analysis. Finally, the GTWR method was employed to establish spatiotemporal relationship models between PM2.5 and explanatory variables, analyzing the spatiotemporal distribution characteristics and driving factors of GTWR.

#### 2.3.1 Spatial autocorrelation analysis

In urban thermal environment research, spatial autocorrelation is a method used to describe the spatial correlation of geographic data. Moran’s I is a commonly used spatial autocorrelation statistic, measuring the overall spatial correlation degree of geographical data [43]. It can be divided into global Moran’s Index and local Moran’s Index. The calculation formulas are as follows:

$$Global\;Moran^{\prime }s\;I=\frac{n\sum_{i=1}^{n}\sum_{j\neq i}^{n}W_{ij}\left(x_{i}-\bar{x}\right)\left(x_{j}-\bar{x}\right)}{\sum_{i=1}^{n}\sum_{j\neq i}^{n}W_{ij}\sum_{i=1}^{n}{\left(x_{i}-\bar{x}\right)}^{2}}$$ (1)

$$Local\;Moran^{\prime }s\;I=\frac{n\left(x_{i}-\bar{x}\right)\sum_{j=i}^{n}W_{ij}\left(x_{j}-\bar{x}\right)}{\sum_{i=1}^{n}{\left(x_{i}-\bar{x}\right)}^{2}}$$ (2)

Where *n* is the number of geographic units; *x*_*i*_ is the observed value of the *ith* geographic unit; $\bar{x}$ is the mean value of all geographic units; *W*_*ij*_ is the spatial weight between geographic unit *i* and geographic unit *j*. The Moran’s Index ranges from -1 to 1, where positive values indicate positive correlation and negative values indicate negative correlation. Values close to 1 or -1 suggest significant spatial clustering patterns.

Local Indicators of Spatial Association (LISA) aggregation analysis is used to detect local spatial clustering patterns in geographic space. By calculating the Moran’s Index between each geographic unit and its neighboring units, geographic units with significant spatial clustering or dispersion patterns can be identified. LISA aggregation analysis produces four quadrants: High-High, Low-Low, High-Low, and Low-High. In the High-High and Low-Low quadrants, the observed values of geographic units exhibit clustering patterns, while in the High-Low and Low-High quadrants, they show dispersion patterns. This helps understand the local clustering characteristics in spatial distribution.

#### 2.3.2 Geographically and temporally weighted regression model

GWR is a regression analysis method used for spatial data. Unlike traditional global regression models, GWR allows the model’s parameters to vary spatially, better capturing heterogeneity and local correlations in geographic spatial data [44]. The regression model of GWR can be expressed as:

$$Y_{i}=\beta_{i}^{0}+\sum_{k=1}^{P}\beta_{i}^{k}X_{i}^{k}+\varepsilon_{i}$$ (3)

Where *Y*_*i*_ is the observed value of the dependent variable at geographic location *i*; *P* corresponds to the number of independent variables; $X_{i}^{k}$ is the observed value of independent variable *k* at geographic location *i*; $\beta_{i}^{0}$ and $\beta_{i}^{k}$ are the intercept and regression coefficient at geographic location *i*; *ε*_*i*_ is the error term.

The key point of GWR is the use of different regression coefficients for each geographic location. The spatial weight matrix reflects the variation of spatial dependence within the study area. This allows GWR to have the ability to detect spatial heterogeneity and improve the accuracy of regression fitting. GWR generally uses a specific distance decay function to determine the elements of the weight matrix, and the spatial bandwidth controls the decay rate. Samples closer in distance have larger weights, while those farther away have smaller weights.

The GTWR model is a statistical model used to handle spatiotemporal data, combining GWR and spatiotemporal modeling methods. Utilizing the GTWR model enables the detection of local regression coefficients of PM2.5 and its influencing factors, meeting the quantitative analysis requirements for spatiotemporal correlations between PM2.5 concentrations and driving factors [45]. Similar to GWR in addressing spatial variations, the GTWR model constructs time and spatial weight matrices based on time and spatial distances. The general formula of the GTWR model can be represented as follows:

$$Y_{i}=\beta^{0}\left(u_{i},v_{i},t_{i}\right)+\sum_{k=1}^{P}\beta^{k}\left(u_{i},v_{i},t_{i}\right)X_{i}^{k}+\varepsilon_{i}$$ (4)

Where (*u*_*i*_, *v*_*i*_, *t*_*i*_) denotes the coordinates of object i in space (*u*_*i*_, *v*_*i*_) at time *t*_*i*_, *β*^0^ (*u*_*i*_, *v*_*i*_, *t*_*i*_) represents the intercept coefficient, *β*^*k*^ (*u*_*i*_, *v*_*i*_, *t*_*i*_) indicates the coefficients of independent variables $X_{i}^{k}$ (*p* in total), and *ε*_*i*_ represents the random error term.

The GTWR model considers the temporal and geographical characteristics of spatiotemporal data and constructs time and spatial weight matrices based on the spatiotemporal autocorrelation and heterogeneity of the data, employing different weights at each spatiotemporal point to model the regression relationship. The spatiotemporal modeling of the GTWR model considers the temporal and spatial changes and dependencies of data, enhancing the modeling accuracy of spatiotemporal data and providing more reliable spatiotemporal predictions and analyses, thereby expanding the methods for spatiotemporal data analysis [46].

## 3. Results

### 3.1 Temporal and spatial characteristics

**Fig 2:**
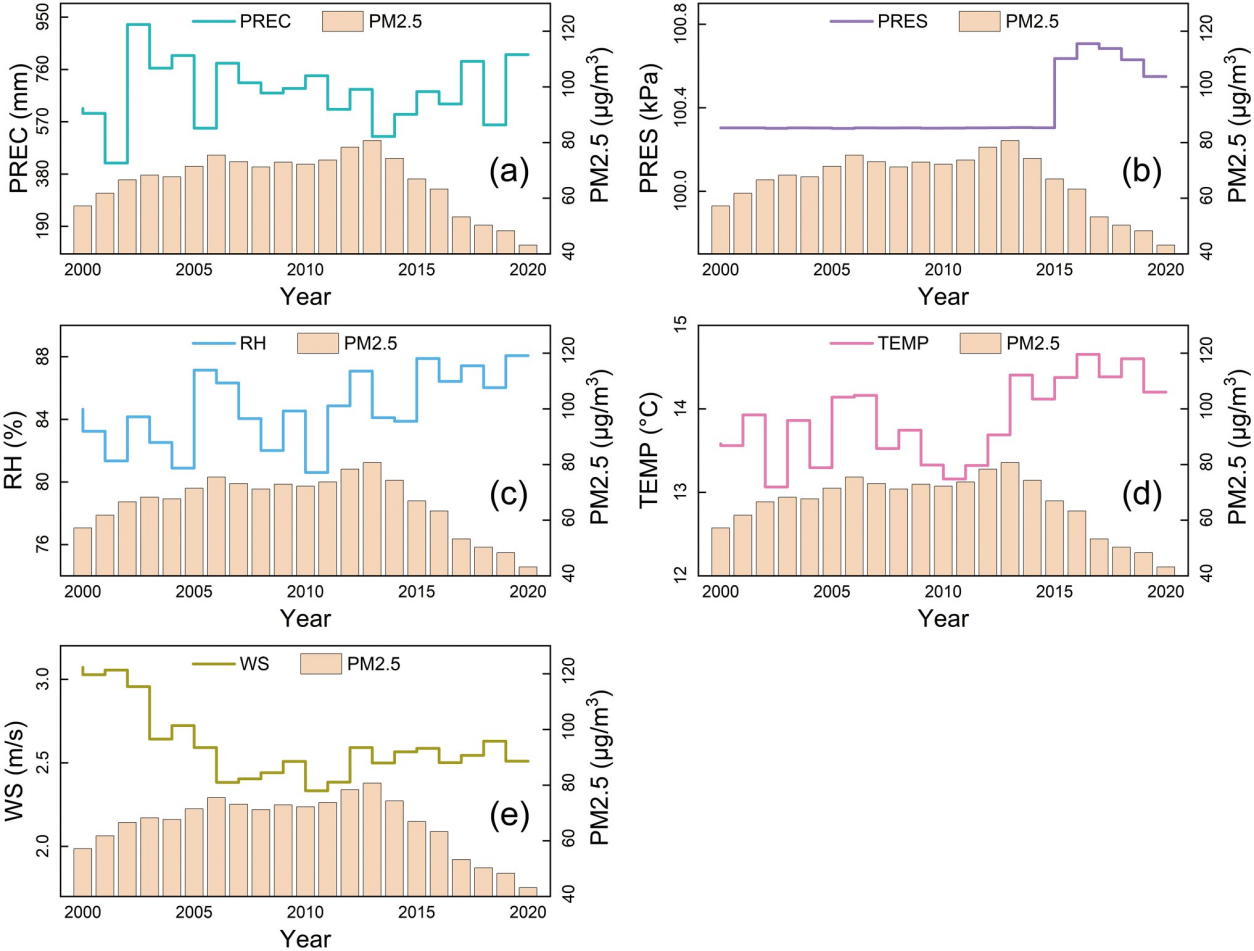
Overall variations of meteorological factors from 2000 to 2020.

**Fig 3:**
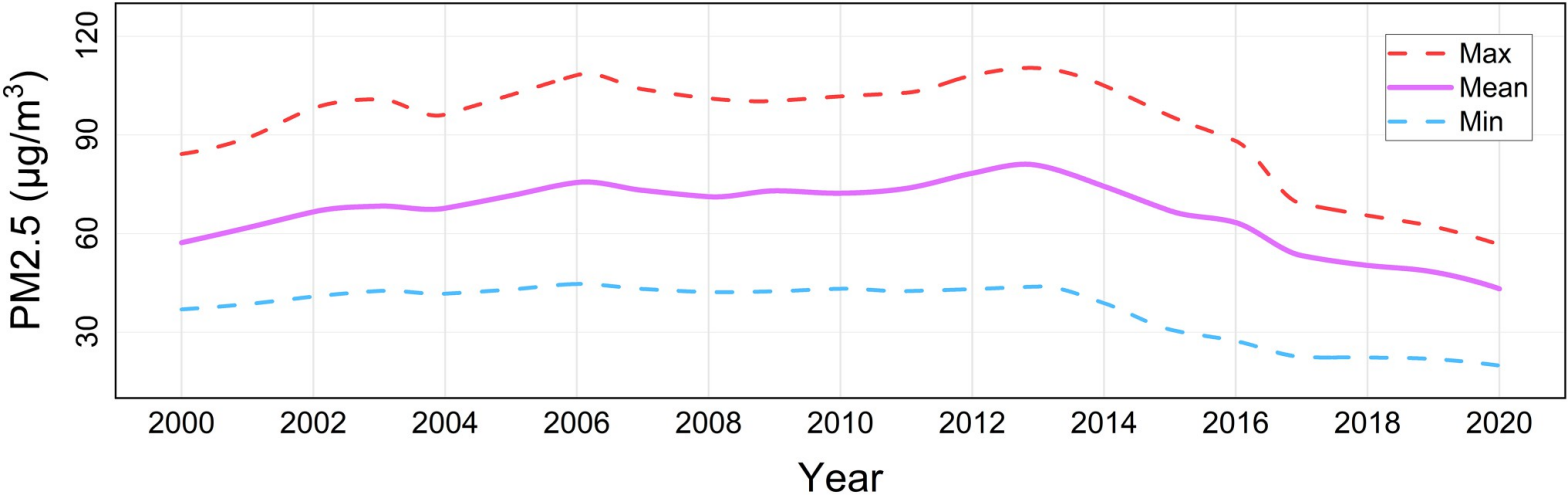
Temporal variation of annual average PM2.5 concentration and extremes in Shandong Province from 2000 to 2020.

The temporal variations of PM2.5 concentration and meteorological factors during 2000–2020 are depicted in Fig 2. Among them, precipitation, relative humidity, temperature, and wind speed exhibit significant fluctuations, while atmospheric pressure shows pronounced fluctuations after 2015. Fig 3 illustrates the temporal variations in the annual average concentration of PM2.5 and its extrema in Shandong Province from 2000 to 2020. The results reveal a trend of initially increasing and then rapidly decreasing PM2.5 concentration. Specifically, this period can be divided into three stages: a fluctuating upward stage, a peak fluctuation stage, and a rapid decline stage. In the first stage (2000–2006), the annual average concentration of PM2.5 increased from 57.22 μg/m^3^ to 75.51 μg/m^3^, with an average growth rate of 5.32%. In the second stage (2006–2013), PM2.5 concentration experienced a brief trough in 2008 at 71.18 μg/m^3^, slightly below the mean of 74.71 μg/m^3^. In the third stage (2013–2020), PM2.5 concentration rapidly decreased from 80.0 μg/m^3^ in 2013 to 43.16 μg/m^3^ in 2020. This evolution is highly likely associated with government policies, technological advancements, industrial restructuring, and increased environmental awareness. The global economic slowdown due to the 2008 financial crisis reduced energy and resource consumption, thus partially controlling the deterioration of air pollution. Air quality gradually became the focal point of environmental protection efforts, leading Shandong Province to officially embark on an eight-year journey towards clear skies in 2013 and to enact the "Shandong Province Atmospheric Pollution Prevention and Control Plan (2013–2020)". Subsequently, regional air pollutant emission standards were phased in gradually. In 2020, industrial pollution privileges related to air pollution were officially abolished. Especially during the third stage, the meteorological factors show strong correlation changes, which side by side indicates that non-meteorological factors indirectly affect the air quality level by improving the meteorological factors.

**Fig 4:**
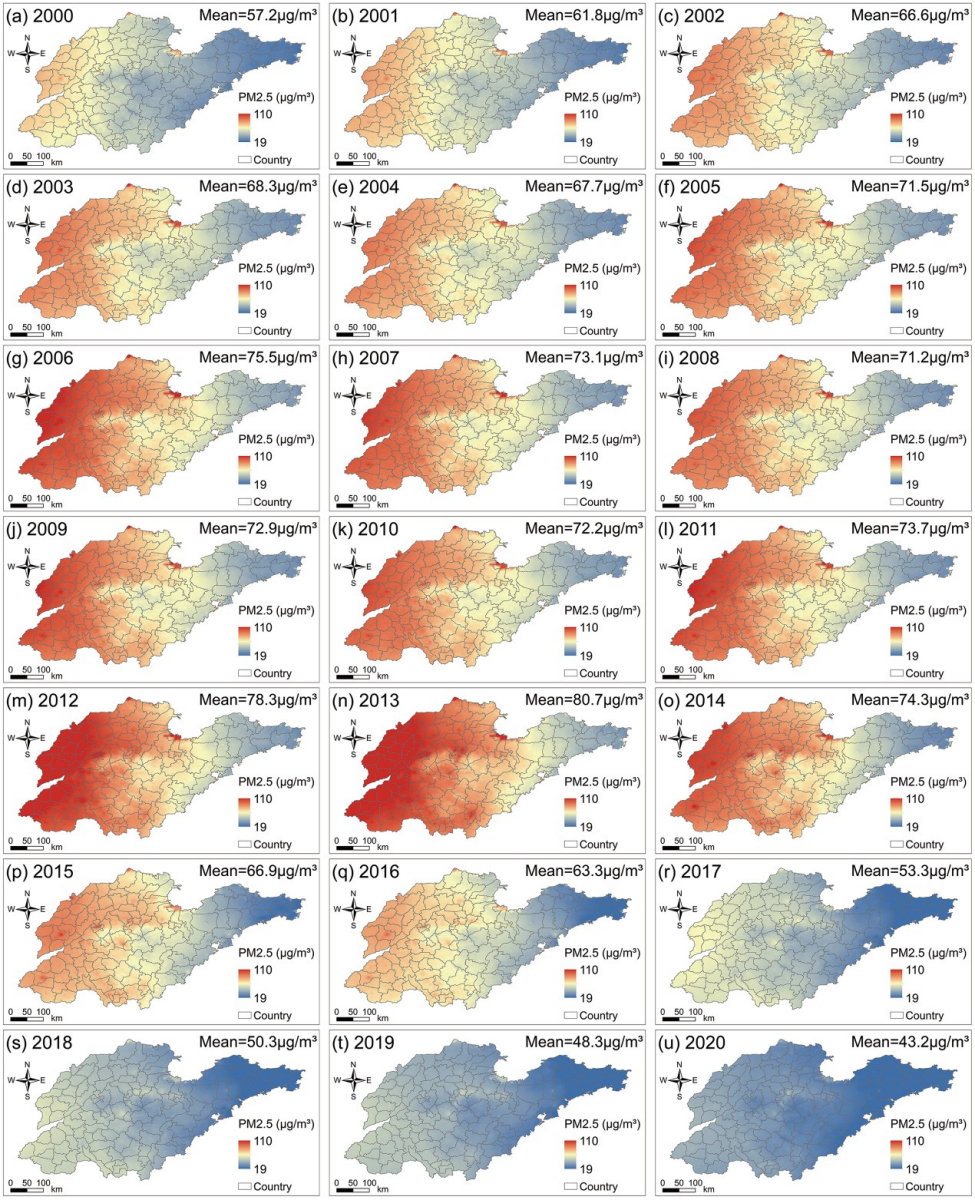
Spatial distribution of PM2.5 average concentrations in Shandong Province from 2000 to 2020.

Fig 4 lists the spatial distribution of annual average PM2.5 concentrations in Shandong Province from 2000 to 2020. The results indicate that areas with the highest PM2.5 concentrations (>71 μg/m^3^) are mainly located in the western plain areas of Shandong, including Liaocheng, Heze, Dezhou, and Jining. Cities in central (such as Jinan, Taian, and Weifang), southern (such as Zaozhuang and Linyi) and northern (such as Binzhou and Dongying) Shandong have relatively high PM2.5 concentration levels, ranging from 57 μg/m^3^ to 70 μg/m^3^. Regions with lower PM2.5 concentrations are mainly concentrated in the Jiaodong Peninsula, including Weihai, Yantai, and Qingdao. Among them, Guanxian, Shenxian, and Linqing in Liaocheng City have the highest PM2.5 concentrations, at 83.69 μg/m^3^, 82.83 μg/m^3^, and 82.50 μg/m^3^, respectively, while Rongcheng, Wendeng, and Huancui in Weihai City have the lowest PM2.5 concentrations, at 41.40 μg/m^3^, 41.92 μg/m^3^, and 42.03 μg/m^3^, respectively.

**Fig 5:**
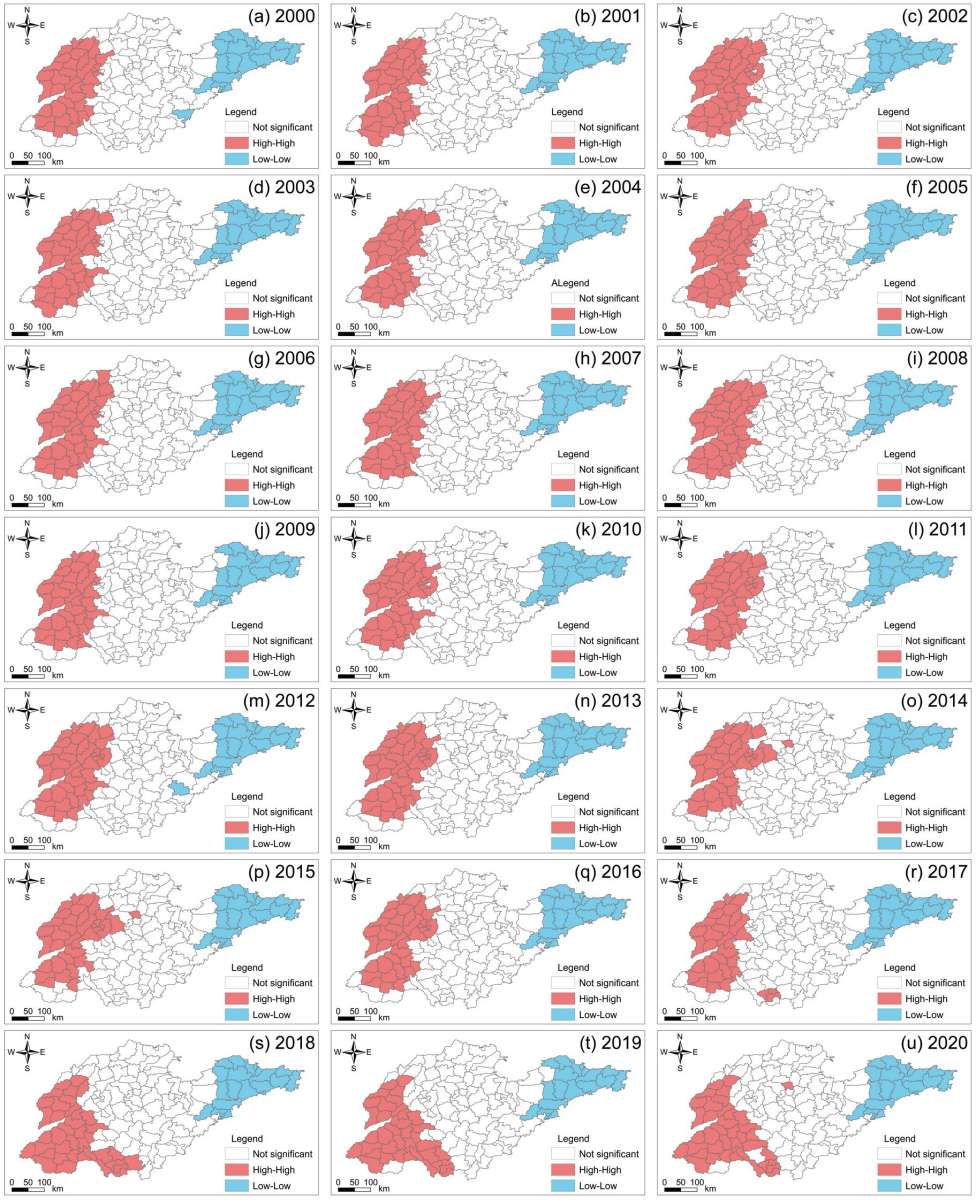
Local Indicators of Spatial Association (LISA) results for PM2.5 spatial autocorrelation analysis in Shandong Province from 2000 to 2020.

This study further employed spatial autocorrelation analysis techniques to diagnose the local spatial clustering of PM2.5 concentrations (Fig 5). The results indicate a clustering convergence trend of PM2.5 concentrations, characterized by significant low-low clusters and high-high clusters. Moreover, the spatial clustering trend of PM2.5 concentrations did not undergo significant changes from 2000 to 2020. High-high clusters are distributed in the western plain areas, including Liaocheng, Heze, Dezhou, and Jining Cities. This indicates that these regions have a high level of air pollution, with extremely strong spatial correlation of PM2.5 pollution across counties. Cities with lower PM2.5 concentrations are mainly concentrated in the Jiaodong Peninsula, such as Weihai, Yantai, and Qingdao, indicating relatively low levels of air pollution and weaker spatial correlation of PM2.5 pollution among counties.

### 3.2 Factors influencing PM2.5 concentrations

To accurately explore the potential relationship between PM2.5 concentrations and influencing factors, this study employed Ordinary Linear Regression (OLR), GWR, and GTWR. Table 3 presents the statistical analysis results of the three regression models, indicating that GTWR is the most suitable for studying the spatiotemporal evolution patterns of PM2.5 concentrations. In the results, the adjusted residual squares (adjR^2^) of OLR is 0.437, which is increased to 0.892 by GWR and further increased to 0.936 by GTWR. This indicates that the GTWR model can explain over 90% of the variation in PM2.5 concentrations, thus enabling the exploration of the impact of meteorological elements and natural socioeconomic factors on PM2.5 concentrations.

**Table 3:** Statistical analysis parameters of PM2.5 in Shandong Province (OLR, GWR, GTWR).

Statistic	OLR	GWR	GTWR
Bandwidth	-	0.12	0.12
Residual squares	70885.21	13483.23	2968.74
Sigma	-	4.44	2.08
AICc	-	4193.16	3337.53
R squares (R^2^)	0.439	0.893	0.937
Adjusted R squares (adjR^2^)	0.437	0.892	0.936
Spatiotemporal distance ratio	-	-	1.16
Trace of S matrix	-	88.75	149.98

#### 3.2.1 Meteorological elements affecting PM2.5 concentrations

**Fig 6:**
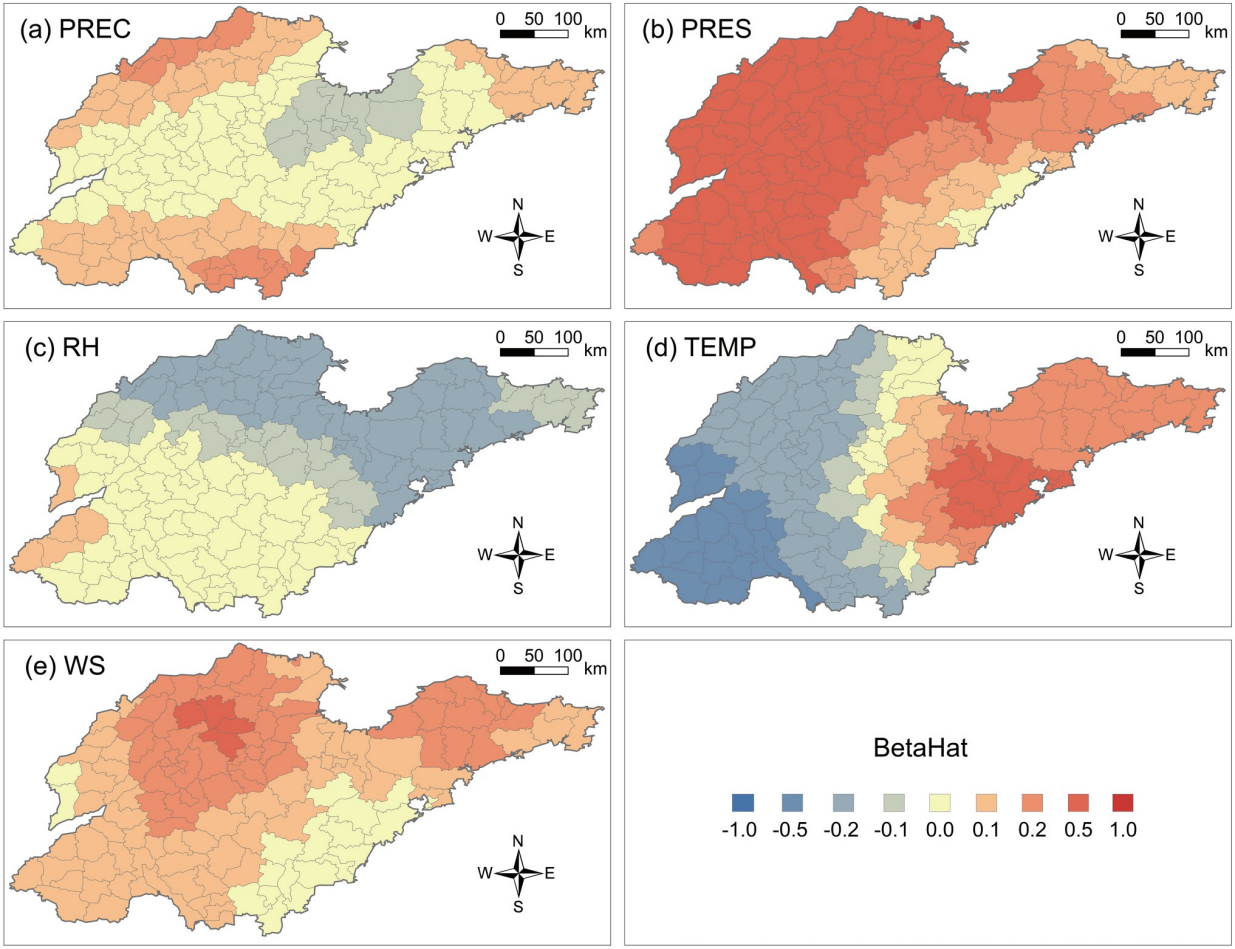
GTWR regression coefficient results for meteorological elements of 136 counties in Shandong Province. (a) Average precipitation (PREC); (b) Average atmospheric pressure (PRES); (c) Relative humidity (RH); (d) Average temperature (TEMP); (e) Average wind speed (WS).

Fig 6(a) provides detailed information on the precipitation coefficients of 136 counties in Shandong Province. It can be observed that precipitation in the southern part of Laizhou Bay and central Shandong is negatively correlated with PM2.5 concentrations, indicating a decrease in PM2.5 concentrations with increasing precipitation in these areas. Conversely, in northern, southern, and the Jiaodong Peninsula regions, precipitation is positively correlated with PM2.5 concentrations, indicating an increase in PM2.5 concentrations with increasing precipitation. Notably, the mitigation effect of PM2.5 concentrations is most pronounced in Guanxian, Changyi, and Weicheng districts, with coefficients of -0.145, -0.136, and -0.133, respectively.

Fig 6(b) shows a gradual decrease in the correlation between PM2.5 concentrations and atmospheric pressure as one moves from northwest to southeast Shandong. Except for some counties in the southeast and the Jiaodong Peninsula with negative coefficients, counties in northern, western, and central Shandong have positive coefficients. These empirical results indicate that an increase in atmospheric pressure has an inhibitory effect on PM2.5 concentrations in parts of the southeast and the Jiaodong Peninsula, while it exacerbates PM2.5 pollution in most areas of Shandong. Notably, Lanshan district exhibits the most significant mitigating effect of atmospheric pressure on PM2.5 concentrations, with a coefficient of -0.019, followed by Donggang district at -0.017 and Huangdao district at -0.009.

Fig 6(c) displays the spatial distribution of relative humidity coefficients in Shandong Province. It can be observed that the areas with the highest correlation between relative humidity and PM2.5 concentrations are mainly concentrated in the southwest of Shandong, including Juancheng county (0.021), Dongming county (0.018), and Mudan district (0.012). Additionally, in the southern region of Shandong, regions such as Shenxian county (0.009), Lanshan district (0.009), and Fei county (0.007) exhibit a positive correlation between relative humidity and PM2.5 concentrations. Conversely, there is a significant negative correlation between relative humidity and PM2.5 concentrations in counties such as Lijin county (-0.474), Hekou district (-0.452), and Wudi county (-0.394), indicating a strong mitigating effect of relative humidity on PM2.5 concentrations.

Fig 6(d) demonstrates the correlation between temperature and PM2.5 concentrations across Shandong Province. As one moves from southwest to southeast Shandong, the correlation between temperature and PM2.5 concentrations gradually shifts from negative to positive. The trend resembles a clear hierarchical structure, with a negative core appearing in Heze in the southwest and a positive core at the intersection of Qingdao, Weifang, and Rizhao in the southeast. Apart from the Jiaodong Peninsula region, most areas in Shandong have positive coefficients. This suggests that an increase in temperature in counties in southwestern Shandong inhibits PM2.5 concentrations, while an increase in temperature in counties in the Jiaodong Peninsula exacerbates air pollution. Notably, Dongming county exhibits the greatest mitigating effect of temperature on PM2.5 concentrations, with a coefficient of -0.707, followed by Mudan district at -0.672 and Cao county at -0.671. Conversely, the temperature coefficient in Huangdao district is 0.299, followed by Zhucheng district (0.261) and Jiaozhou district (0.253).

Fig 6(e) illustrates the spatial distribution of wind speed coefficients and their correlation with PM2.5 concentrations in Shandong Province, indicating a gradual weakening of correlation as one moves from the north to the south. There is a significant positive correlation between wind speed and PM2.5 concentrations in northern Shandong and the Jiaodong Peninsula region. Counties with a negative correlation with PM2.5 concentrations are mainly located in the southeast, including Rizhao and Linyi, as well as in the western plain areas. The experimental results demonstrate that Lanshan district exhibits the strongest negative correlation between wind speed and PM2.5 concentrations, with a coefficient of -0.088, followed by Donggang district (-0.083) and Junan county (-0.069). Huimin county has the greatest positive impact on PM2.5 concentrations, with a coefficient of 0.209, followed by Zouping city (0.203) and Gaoqing county (0.202).

#### 3.2.2 Natural socioeconomic factors influencing PM2.5 concentrations

**Fig 7:**
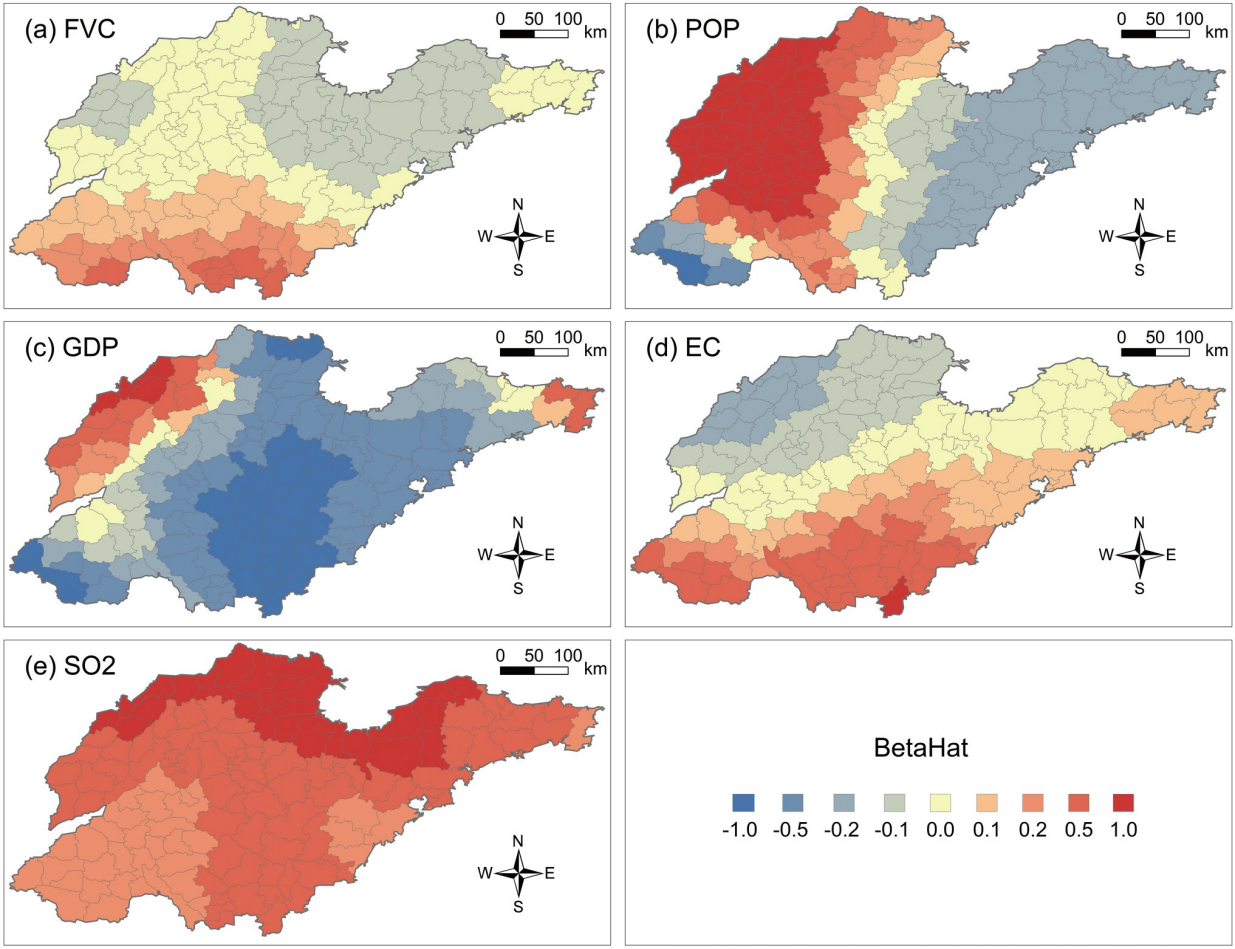
GTWR regression coefficient results for natural socioeconomic conditions of 136 counties in Shandong Province. (a) Fractional vegetation cover (FVC); (b) Population density (POP); (c) GDP density (GDP); (d) Electricity consumption (EC); (e) SO2 emissions (SO2).

In terms of natural factors, FVC generally inhibits the increase and diffusion of PM2.5 concentrations. The FVC coefficients show an increasing trend from north to south in Shandong Province (see Fig 7(a)). In southern Laizhou Bay, most counties in Weifang and Yantai, FVC significantly mitigates air pollution. In counties in southern and southwestern Shandong, the positive correlation between FVC and PM2.5 concentrations is more pronounced compared to other regions. Notably, the coefficient in Laizhou city (-0.167) is the largest among the negative correlation coefficients, followed by Changyi city (-0.166) and Hanting district (-0.162). The coefficients in Zaoyuan city, Kuiwen district, Pingdu city, Longkou city, and Weicheng district are all below -0.150.

Population density (POP) generally makes a significant contribution to the increase in PM2.5 concentrations. From northwest to southeast Shandong, the correlation between population density and PM2.5 concentrations gradually decreases (see Fig 7(b)). Counties in southwestern Shandong and coastal counties in the Jiaodong Peninsula have significantly negative coefficients, indicating that an increase in population density is likely to be accompanied by a continuous decrease in PM2.5 concentrations. The northwest part of Shandong is mostly flat terrain, where PM2.5 concentrations rapidly accumulate with the increase in population density and human activities. Overall, the contribution of population density to PM2.5 concentrations is smaller in southeastern areas (<-0.200), while the increase in human activities in northwestern areas exacerbates environmental pressure (>0.500). Due to the limited carrying capacity of the natural environment, the increase in population density not only accompanies economic development but also exacerbates PM2.5 pollution.

The correlation between GDP and PM2.5 concentrations exhibits obvious spatial differentiation (see Fig 7(c)). GDP correlation coefficients are higher in western Shandong and the Jiaodong Peninsula, exceeding those in central Shandong. It is worth noting that the coefficients in underdeveloped counties in southwestern and northern Shandong are also relatively low. Among the positive correlation coefficients, Ningjin county exhibits the highest correlation, with a coefficient of 0.650, followed by Decheng district (0.598) and Wucheng county (0.529). The lowest negative correlation coefficient is -1.817 in Dongming county, followed by -1.564 in Cao county and -1.415 in Tancheng county. GDP directly reflects the intensity of economic activities in various counties in Shandong Province, with higher GDP levels indicating higher levels of pollutant emissions.

In terms of electricity consumption, industrial electricity consumption accounts for a large proportion, while residential electricity consumption is negligible. In the early stages of economic development in most counties with underdeveloped or structurally unreasonable industries, high-polluting factories were introduced, causing serious damage to local air quality. The correlation between electricity consumption and PM2.5 concentrations gradually strengthens as one moves from northwestern Shandong to southeastern Shandong (see Fig 7(d)). Regions with positive correlations are mainly distributed in southern Shandong, including Tancheng county (0.513), Linsu county (0.435), Lanling county (0.434), as well as in southwestern Shandong, including Cao county (0.447), Dongming county (0.415), and Shan county (0.363). Meanwhile, the counties of Decheng, Wucheng, and Ningjin in northwestern Shandong maintain strong negative correlation relationships (-0.307, -0.303, and -0.288, respectively). Additionally, the coefficients in Rongcheng city, Huancui district, and Wendeng district in the Jiaodong Peninsula all exceed 0.025.

Counties with high SO2 coefficients are distributed in northern Shandong and the Laizhou Bay area, with all counties in Shandong Province showing significant positive coefficients, indicating that PM2.5 concentrations will accelerate with increasing SO2 emissions (see Fig 7(e)). Counties with coefficients exceeding 0.900 include Lijin county, Hekou district, Kenli district, Lijin county, and Zhanshan district. Meanwhile, the coefficients in Rencheng district, Jinxiang county, Yutai county, Yanzhou county, and Jiaxiang county are all below 0.560. Keeping other factors constant, reducing SO2 emissions in regions with high correlation coefficients can significantly reduce PM2.5 concentration levels. Therefore, focusing on controlling the sources of SO2 emissions in the subordinate counties of Laizhou Bay area and Linyi city can effectively address the serious air pollution problem in Shandong Province.

## 4. Discussion

This study conducted an analysis of PM2.5 concentrations in Shandong Province from 2000 to 2020 at the county level and investigated the spatiotemporal variations of driving factors for PM2.5 concentrations using the GTWR model. We observed significant temporal and spatial heterogeneity in both PM2.5 concentration levels and their determining factors. In comparison with traditional research focuses, the findings of this study contribute to the precise identification of meteorological elements and socioeconomic conditions that influence the occurrence and intensity of air pollution events.

The impact patterns of meteorological elements on PM2.5 concentrations vary. Precipitation primarily reduces air pollution levels in the surrounding Laizhou Bay and central Shandong regions. When precipitation reaches a certain level, it has a cleansing effect on particulate matter in the air; however, excessively light precipitation can exacerbate air pollution. An increase in relative humidity significantly reduces the formation of PM2.5 pollutants, particularly evident in northern and surrounding Laizhou Bay areas of Shandong. Changes in air pressure are closely related to temperature, with opposite effects on PM2.5 concentrations. Higher temperatures in western Shandong are conducive to improving air quality by enhancing urban heat island effects and air convection rates, facilitating the rapid dispersion of air pollutants generated in urban areas to suburban areas. Although air movement aids in the transfer and dispersion of pollutants, its alleviating effect on PM2.5 pollution in Shandong Province is not significant due to topographical constraints [47]. During winter, haze brought by northwest winds accumulates in northern Shandong, while the northern part of the Jiaodong Peninsula faces the Bohai Sea, where the condensation of seawater retains heavy pollutants from northeastern China.

The natural factor FVC reflects the ecological environment’s quality in Shandong Province. It significantly mitigates PM2.5 pollution levels in the southern part of surrounding Laizhou Bay and northwestern Shandong but exacerbates pollution in southern and southwestern Shandong. The western plains of Shandong accommodate most of the population, leading to severe resource consumption and air pollution issues. However, this situation improves in some counties in southwestern Shandong. Residents’ economic income in western Shandong and the Jiaodong Peninsula is better than in central, southern, and southwestern Shandong, and GDP accurately reflects the economic vitality of these regions. Areas with higher GDP exhibit a stronger positive correlation with PM2.5 concentrations, while areas with lower GDP make minimal contributions to PM2.5 concentrations. As southern and southwestern Shandong primarily rely on agriculture and have weak economic foundations and limited development space, they attract high-energy-consuming and high-polluting industries phased out by other advanced regions [48], posing a severe threat to regional air quality. Higher levels of electricity consumption exacerbate PM2.5 levels. As a direct source of air pollutants, SO2 maintains a high positive correlation with PM2.5 concentrations [49], especially in the surrounding Laizhou Bay area.

In contrast to meteorological elements that cannot be altered, proactive measures can be taken to adjust natural and socioeconomic factors, actively addressing a series of ecological environment security and public health issues caused by PM2.5 pollution. Implementable measures include: (1) prioritizing environmental pollution issues and enhancing environmental protection policies and measures, such as strengthening industrial pollution control, promoting the development of clean energy, and restricting automobile exhaust emissions; (2) emphasizing technological innovation and upgrading pollution control technology levels by introducing new environmental protection technologies and equipment, such as more efficient industrial filters and clean energy technologies, to reduce pollutant emissions at the source; (3) accelerating industrial restructuring and actively transitioning to cleaner industries by reducing the proportion of high-pollution and high-emission industries and increasing the proportion of clean production to reduce total pollutant emissions; (4) fostering environmental awareness and advocating for public participation in environmental protection actions. Increased public awareness of air quality issues and enhanced support and participation in environmental protection actions can prompt governments and enterprises to prioritize environmental protection work.

In investigating the spatiotemporal patterns of county-scale PM2.5 drivers in Shandong Province, several methodological and analytical limitations and uncertainties must be acknowledged: (1) Our reliance on satellite-derived PM2.5 data, while providing extensive coverage, comes with limitations in spatial and temporal resolution. These limitations might obscure finer-scale variations and lead to potential inaccuracies in the dataset; (2) The GTWR model, though effective in capturing local heterogeneity, demands substantial computational resources and optimal bandwidth selection. Moreover, the model may not fully capture nonlinear dependencies among variables, potentially oversimplifying the relationships between PM2.5 concentrations and their drivers; (3) Shandong Province’s diverse topography, climate, and economic conditions can lead to varying PM2.5 dynamics that are not entirely accounted for by the model. While GTWR addresses some spatial variability, it might still overlook localized extreme conditions and unique regional characteristics; (4) Although our study indicates the effectiveness of air pollution control measures, the temporal and regional variability in policy implementation and enforcement is difficult to model accurately. Other unmodeled anthropogenic factors, such as industrial shifts and technological advancements, also contribute to uncertainties in understanding PM2.5 trends.

For future research, several directions can enhance the understanding of PM2.5 dynamics and their drivers in Shandong Province. Firstly, improving data accuracy and resolution is crucial. Integrating higher-resolution satellite data with ground-based monitoring can provide more precise and detailed PM2.5 concentration maps. Additionally, employing advanced data assimilation techniques can mitigate calibration errors and enhance dataset reliability. Secondly, enhancing the GTWR model to account for nonlinear interactions and dependencies among variables would provide a more comprehensive analysis. Incorporating machine learning algorithms, such as random forests or neural networks, can help capture complex relationships and improve predictive accuracy. Thirdly, addressing the spatiotemporal bandwidth’s diversification more effectively is essential. We plan to involve the utilization of the more advanced Multiscale Geographically and Temporally Weighted Regression Model (MGTWR) [50] to detect unique spatiotemporal bandwidths for different variables, thereby expressing the spatiotemporal heterogeneity of PM2.5 driving factors more accurately. Fourthly, incorporating a broader range of anthropogenic factors, such as industrial relocation, technological advancements, and urban planning policies, would provide a more holistic view of PM2.5 drivers. Longitudinal studies tracking these factors over time could elucidate their impacts on air quality more clearly.

In conclusion, this study reveals the spatiotemporal variations of PM2.5 concentrations and their potential influencing factors by examining the spatiotemporal evolution of driving factors. The empirical analysis at the county level in Shandong Province fills the gaps in existing literature and provides valuable insights for air pollution control in counties with different development levels. Due to significant spatiotemporal variations in the relationships between PM2.5 concentrations and meteorological elements as well as natural socioeconomic conditions, different counties in Shandong Province face distinct air pollution issues. When formulating pollution reduction and control measures, the unique meteorological and natural socioeconomic conditions of each county must be considered first. The spatiotemporal changes and dispersion of PM2.5 pollution influenced by various factors necessitate environmental management departments to consider county-level differences in formulating targeted policies.

## 5. Conclusion

This research offers a pioneering analysis of the spatiotemporal patterns of PM2.5 drivers at the county level in Shandong Province over a two-decade span from 2000 to 2020. Utilizing the GTWR, the research innovatively integrates meteorological elements and natural-socioeconomic conditions to identify key factors influencing PM2.5 concentrations across 136 counties. The significant findings are as follows: (1) PM2.5 pollution in Shandong Province peaked in 2013, followed by a rapid decline, showcasing the effectiveness of recent pollution control measures; (2) The study highlights distinct geographical disparities in PM2.5 concentrations, which underscores the need for region-specific pollution control strategies; (3) The statistical results identify the dual role of drivers in PM2.5 dynamics, which have a positive impact in some areas and show a negative impact in others; (4) The findings support the effectiveness of existing air pollution control measures and provide a quantitative basis for refining regional emission reduction policies. The spatiotemporal heterogeneity of PM2.5 pollution necessitates tailored strategies that consider the unique environmental and socioeconomic conditions of each county. The paper’s innovative use of the GTWR model to analyze county-level data over an extended period represents a significant methodological advancement. Future research will enhance this approach by incorporating the MGTWR to further refine the understanding of spatiotemporal heterogeneity in PM2.5 drivers. In summary, this research provides a comprehensive and nuanced understanding of the factors driving PM2.5 pollution in Shandong Province, offering valuable insights for targeted environmental management and policy formulation at the county level.
